# Three-Dimensional Multi-Modality Registration for Orthopaedics and Cardiovascular Settings: State-of-the-Art and Clinical Applications

**DOI:** 10.3390/s24041072

**Published:** 2024-02-07

**Authors:** Simone Garzia, Katia Capellini, Emanuele Gasparotti, Domenico Pizzuto, Giuseppe Spinelli, Sergio Berti, Vincenzo Positano, Simona Celi

**Affiliations:** 1 BioCardioLab, Bioengineering Unit, Fondazione Toscana G. Monasterio, 54100 Massa, Italy; sgarzia@ftgm.it (S.G.); kcapellini@ftgm.it (K.C.); gasparotti@ftgm.it (E.G.); positano@ftgm.it (V.P.); 2 Department of Information Engineering, University of Pisa, 56122 Pisa, Italy; dompizz.95@gmail.com; 3 Maxillofacial Surgery Department, Azienda Ospedaliero-Universitaria Careggi, 50134 Firenze, Italy; spinellig@aou-careggi.toscana.it; 4 Diagnostic and Interventional Cardiology Department, Fondazione Toscana G. Monasterio, 54100 Massa, Italy; ifcberti@ftgm.it

**Keywords:** multimodal registration, 3D Slicer, landmark, orthopaedic, cardiovascular

## Abstract

The multimodal and multidomain registration of medical images have gained increasing recognition in clinical practice as a powerful tool for fusing and leveraging useful information from different imaging techniques and in different medical fields such as cardiology and orthopedics. Image registration could be a challenging process, and it strongly depends on the correct tuning of registration parameters. In this paper, the robustness and accuracy of a landmarks-based approach have been presented for five cardiac multimodal image datasets. The study is based on 3D Slicer software and it is focused on the registration of a computed tomography (CT) and 3D ultrasound time-series of post-operative mitral valve repair. The accuracy of the method, as a function of the number of landmarks used, was performed by analysing root mean square error (RMSE) and fiducial registration error (FRE) metrics. The validation of the number of landmarks resulted in an optimal number of 10 landmarks. The mean RMSE and FRE values were 5.26 ± 3.17 and 2.98 ± 1.68 mm, respectively, showing comparable performances with respect to the literature. The developed registration process was also tested on a CT orthopaedic dataset to assess the possibility of reconstructing the damaged jaw portion for a pre-operative planning setting. Overall, the proposed work shows how 3D Slicer and registration by landmarks can provide a useful environment for multimodal/unimodal registration.

## 1. Introduction

Medical imaging plays a pivotal role in the diagnosis, treatment planning, and monitoring of various medical diseases and conditions. In clinical practice, different imaging modalities, such as computed tomography (CT), magnetic resonance imaging (MRI), and ultrasound (US), are employed to capture different aspects of anatomical structures and physiological functions. Indeed, multi-modality registration techniques enable the fusion of anatomical information from CT scans, soft tissue details from MRI, and real-time guidance from intra-operative US, enhancing surgical accuracy and reducing patient risk [1,2]. However, each modality possesses its strengths and limitations, making it crucial to integrate and align information from multiple imaging sources to comprehensively understand complex medical scenarios. This integration is achieved through a process called multi-modality registration, which aligns images acquired from different medical imaging modalities into a common reference frame. Formally, the image registration problem can be considered as an optimization problem, aiming to maximize a similarity function that defines alignment quality, also called the *similarity metric.* The search space for possible alignments is defined by a *geometrical transformation*. The algorithm used to maximize the similarity metric is called the *search strategy*. Important aspects in the choice of geometric transformation are the dimensionality of image data (i.e., 2D-2D, 3D-3D, or 2D-3D) and the transformation nature (i.e., global or elastic) [3]. Focusing on dimensionality, 2D-2D registration usually shows a lower complexity, easier implementation, and faster execution than 3D-3D, concerning the amount of data and the number of parameters. Regarding the global transformation, all the points in an image are transformed with the same matrix composed of 12 unknown parameters (nine for roto-translation, one for zooming, and two for sharing). In this context, the rigid transformation is a particular case of a global transformation where zooming and sharing are not allowed. Elastic transformation is a transformation involving each image pixel independently. Thus, the degrees of freedom in the transformation are very high, increasing with the number of pixels. The similarity metric also has a central role in image registration. It is a measure used to evaluate the alignment or similarity between two images during the registration process. Several types of registration similarity metrics are commonly used, including the Sum of Squared Differences (SSD), Mutual Information (MI) [4], and Hausdorff distance [5]. Two different approaches used in image registration are feature-based and voxel-based registration. The feature-based registration is based on a limited set of identifiable landmark points, the alignment of segmented binary structures (typically object surfaces), or direct measurements computed from the image’s grey values. These approaches allow for registration without needing external markers or objects [6]. Instead, the voxel-based metric operates directly on the grey levels of the image, theoretically needing no user intervention or segmentation; the intensity patterns in each image are matched using mathematical or statistical criteria such as SSD, normalized correlation, and MI. Since the adoption of different imaging techniques during interventional procedures continues to progress, the importance of multimodal fusion is growing as well [7,8]. This is particularly true in the case of cardiovascular patients where the biological structures are affected by a significant dynamic movement determined by the heartbeat, and where different sources of images are used for complementary morphological and/or functional investigation [9]; functional and structural data of the medical images are combined to produce more valuable information. In this scenario, the investigation of the heart valve plays a pivotal role in the structural intervention field. However, despite the importance of multimodality imaging techniques in valve treatment [10], attention was focused mainly on the aortic valve district [11], and only a few works were presented regarding CT/US registration approaches for the mitral valve district [12].

The investigations within this study contribute to the research field by presenting a multimodal image-registration workflow for CT and US scans for mitral valve functionality evaluation in a clinical setting. A second case study is also presented, focusing on unimodal images to evaluate the feasibility of our approach in a clinical orthopaedic scenario.

The manuscript is structured as follows: Firstly, an overview of the state-of-the-art in multimodality registration techniques in cardiology (Section 2.1) and orthopaedics (Section 2.2) is presented. Then two specific case studies based on landmark registration techniques for both the cardiovascular (Section 3.1) and orthopaedic fields (Section 3.2) are described. Section 4 presents the outcomes of both case studies, while Section 5 reports a Discussion and the Conclusions of this work.

## 2. The State-of-the-Art in Multimodality Registration

### 2.1. Cardiovascular Applications

Image registration in interventional cardiology is frequently involved in non-trivial interventions dealing with complex cardiac structures that are deformable by nature. Image registration can be applied in the pre-surgery, intra-surgery, and post-surgery phases. By combining CT, MRI, US, and X-ray fluoroscopy images, clinicians can better understand cardiac anatomy, perfusion, and function. This approach surpasses the efficacy of evaluating separate imaging datasets in isolation or in parallel, as defined in prior studies [13].

A concrete example of the necessity for integration emerged in a detailed study involving 53 patients undergoing Transcatheter Aortic Valve Implantation (TAVI). In this study, the accuracy of assessing the dimensions of the aortic valve annulus using 2D and 3D transesophageal echocardiography was compared with that obtained through CT. It is worth noting that using 2D transesophageal echocardiography to calculate the dimensions of the aortic valve annulus, represented as circular areas, consistently resulted in a significant underestimation, with an average deviation of 16.4% compared to measurements obtained through CT. In contrast, the use of 3D transesophageal echocardiography for dimension assessment yielded a more accurate estimate, with an average underestimation of 9.6% [14].

Multi-modality registration facilitates the alignment of these images, enabling cardiologists to integrate functional data from cardiac MRI with high-resolution anatomical details from CT and real-time imaging from the US. Unfortunately, because of the soft and deformable nature of cardiac structures, automatic registration methods such as rigid registration do not guarantee accurate results, so semi-automatic techniques should be considered. In particular, multimodal image registration in the intra-operative cardiac scenario has recently experienced a surge in attention from the clinical world. In general, it can be performed by a static or dynamic registration. The first can usually involve pre-operative data (i.e., images acquired one at a time, such as the CT ones), which are somehow registered alongside the intraoperative images (i.e., the US). The latter, instead, involves only the intraoperative images acquired during the surgical procedure. Cardiac applications deal mainly with minimally invasive surgery, such as percutaneous interventions or transcatheter valve repair/replacement.

Different image registration approaches involve the use of pre-operative CT as useful data to compensate for the lack of some anatomic information together with post-operative imaging modalities. In these cases, a static registration is often performed. In Ref. [15], a registration framework to align 2D echocardiography images with cardiac CT volume for mitral valve (MV) interventions was developed. The workflow involved temporal and spatial registration steps. The temporal registration aimed to identify echocardiography frames corresponding to the same cardiac cycle phase as the CT volume. Echocardiographic frames were extracted based on the ECG signal, ensuring synchronization with the CT phase. The spatial registration consisted of two steps. In the first step, an expert performed rigid registration by aligning each 2D echocardiography frame with the CT volume using 3D translation, rotation, and 2D scaling. The expert applied this transformation during pre-procedural planning based on CT volume and the patient’s body axes. The second step involved a 2D-3D registration using a rigid intensity-based approach with Normalized-MI (NMI) as the similarity metric. CT data were sampled and interpolated to match the size and spatial location of the echocardiography image. The registration utilized a generalized pattern search algorithm to iteratively find optimal spatial transformation parameters that maximized NMI’s overlap with the image pixel intensities. Once the optimal transformation was found, it was applied to the cardiac CT volume. Validation results demonstrated the promising accuracy of the framework. However, a comparison with the “gold standard” (GS) involved manual rigid transformation by the expert, introducing potential user errors. Useful information can also be retrieved from the registration of specific unimodal medical images with models of target organs deriving from different images. In Ref. [16], the researchers registered intra-operative US images with a dynamic aortic model to improve the performances of a magnetic tracking system. The construction of the dynamic 3D aortic model leverages a pre-operative 4D CT dataset. This dataset’s movement is synchronized with real-time electrocardiograph input from the patient. The automatic extraction of aortic root contours from real-time short-axis US images facilitates the registration process, aligning the 2D intra-operative US image with the dynamic pre-operative aortic model. This registration technique has been proven to be suitable for real-time interventional uses, ensuring precise alignment between the intra-operative 3D model of the aorta and intra-operative US images. The ultimate objective of their study was to improve intracardiac visualization and navigation. More recently, the focus of researchers has shifted to the promising use of neural networks for registration. Over the past few years, a large number of works have been published on medical image registration, differing in the type of images and strategies applied [17,18]. In the case of cardiovascular image registration, for example, in [19], semantic information extracted from a convolutional neural network (CNN) and derived from segmented anatomical labels of 3D cardiac MRI images with local distance metrics for improved alignment of structures were combined. In Ref. [20], the focus of registration was Cardiac Magnetic Resonance images. The objective is reached by exploiting explicitly modeled discontinuities along cardiac boundaries, obtained by slitting cardiac images into four sub-regions, to train a Deep Discontinuity-preserving Image Registration network. Other deep learning based approaches also leveraged Generative Adversarial Networks to generate images with the same characteristics as the floating image both in terms of signal intensity and landmark locations [21,22].

### 2.2. Orthopaedic Applications

Different to cardiovascular structure registration, registration in orthopaedics deals with rigid structures. In the orthopaedic field, the advantages of patch-based rigid image registration algorithms, specifically for improving spinal surgery when using image-guided surgery systems, were explored by ref. [23]. The use of interior-point optimization techniques, specifically, the barrier method, for performing rigid registration was exploited in this work. This approach involved registering CT and US images aiming to enhance the pre-operative assessment and surgical guidance for spinal procedures. The registration process employed three different datasets for vertebrae with corresponding CT, US, and simulated US images and two patch-based rigid image registration algorithms, one based on normalized cross correlation and the other based on the correlation ratio. Both methods focused on correcting the misalignment of the surfaces between the CT and US images, leveraging the complementary information provided by both modalities. Indeed, the registration results demonstrate the effectiveness of the methods in aligning the pre-processed CT and US images, leading to a reduction in the warping index. Based on these findings, the proposed image registration techniques have the potential to enhance ultrasound-guided interventions for spinal procedures.

Multimodality image registration also plays a crucial role in total knee replacement surgeries. A previous study focused on developing a robust and accurate knee joint modelling method to provide precise surgical guidance during knee surgery. To address the challenge of spatial inconsistency caused by knee bending in CT/MRI scans, they employed a multimodality registration strategy that combined MRI and CT images of the femur and tibia separately to overcome this issue. By integrating information from both MRI and CT images, surgeons gained a comprehensive understanding of the patient’s knee anatomy, which is crucial for precise surgical planning and execution [24]. Furthermore, multimodality registration has been applied in orthopedic interventions involving maxillofacial surgeries. In a study conducted by Yu et al. [25], a novel approach for the resection and reconstruction of recurrent maxillary squamous cell carcinoma was presented. A combination of 18F-fluorodeoxyglucose–PET/CT and contrast-enhanced CT was utilized for image fusion. Their methodology involved employing the landmark-based approach provided by Brainlab software to register image series acquired from different modalities. This registration allowed for the precise visualization of critical structures such as vessels, high metabolic tumour regions, nerves, and other vital organs. This approach demonstrated promising potential for improving the accuracy of surgical interventions for recurrent maxillary squamous cell carcinoma, enabling surgeons to better navigate and target the affected areas while minimizing damage to surrounding healthy tissues.

These case studies exemplify the successful implementation of multimodality registration techniques in orthopaedic interventions. Finally, other than spatial criteria, temporal registration in medical images is also a crucial technique that aims to align images acquired at different time points within the same patient. Temporal registration involves finding the spatial transformation that aligns the anatomical structures or functional patterns across the temporal sequence of images. This transformation accounts for changes caused by patient motion, organ deformation, or imaging protocol variations.

Nowadays, similarly to the cardiovascular fields, the state-of-the-art methods for orthopaedics/orthodontics registration are neural networks [26,27]. In the case of orthodontics image registration, for example, Park and colleagues [28] have tried to develop a 3D post orthodontic face prediction method using a deep learning network that incorporates patient-specific factors and orthodontic treatment conditions. To this end, soft tissue masks derived from T1 and T2 CTs were reoriented and registered through stable anatomic structures of the cranial base for extraction and training purposes. The study resulted in an acceptable and naturally appearing whole 3D face as the predicted outcome, in contrast to landmark-based 3D prediction systems, which depend on limited information derived from conventional 2D cephalometric evaluation. An interesting method for measuring in vivo knee joint motion from fluoroscopic images was introduced by Wang et al. [29]. Here, the position and orientation of the knee joints were estimated by tracking selected points with a multiview point-based registration network. This approach involves rapidly registering 2D to 3D data across a wide capture range in combination with a feature-based transfer-learning method that extracts features from fluoroscopic images. Despite having only three subjects and fewer than 100 pairs of real fluoroscopic images, this method achieved good registration results, suggesting a possible solution to limited data availability.

## 3. Materials and Methods

In this study, the application of a landmark-based medical image registration procedure based on freely available 3D Slicer 5.0.2 software is proposed [30]. Landmark-based registration is a semi-automatic image registration approach based on the manual identification of anatomical markers by an expert operator. This approach is flexible and fast, comparable to fully automatic registration. The proposed methods show the effectiveness of the landmark-based approach on two representative clinical cases. All the image processing tasks were performed on a Microsoft Windows 11 operating system workstation with Intel(R)Core(TM) i7-8700K CPU @ 3.70 GHz, 16 GB.

### 3.1. Case Study 1: Landmark Registration in Cardiology

**Figure 1:**
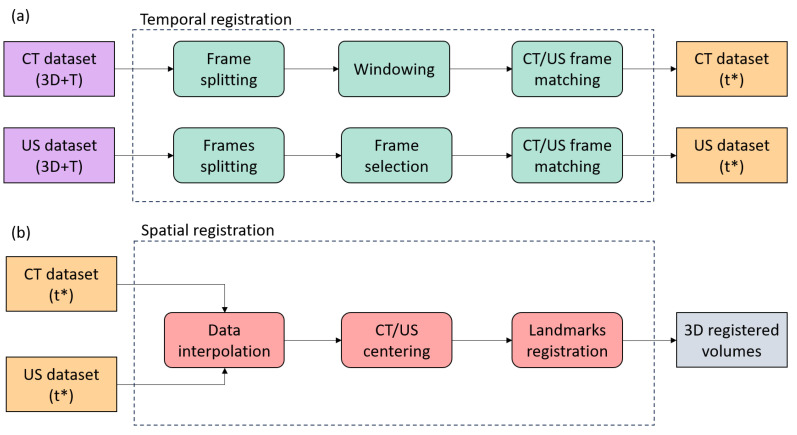
Workflow of the temporal (**a**) and spatial (**b**) registration for the cardiovascular registration.

The first presented clinical case is about the registration of image data acquired by two different imaging modalities, 3D pre-operative CT ($CT_{pre}$) and 3D pre- and post-operative US ($US_{pre}$, $US_{post}$) images, for the assessment of the effectiveness of transcatheter mitral valve intervention by Cardioband^®^ system (Edwards Lifescience, Irvine, CA, USA) [31]. Transcatheter mitral valve implantation (TMVI) allows for a reduction in valve annulus diameter to promote leaflet coaptation. The effectiveness of the procedure can be assessed by comparing the annulus dimension before and after the TMVI procedure by comparison of pre-operative and post-operative 3D US images. High-contrast CT images are used as a “bridge” to reslice with the optimal orientation of the US volumes, finding the best view for annulus measurement. The adopted image registration pipeline is shown in (Figure 1). The pipeline is based on two main steps: a temporal registration to find the corresponding cardiac phases in the two modalities and a spatial rigid landmarks-based registration procedure.

The study population includes 5 patients (3 males and 2 females) with indicated MV functional regurgitation disease who had undergone TMVI between 2016 and 2018. For each patient, a pre-operative, contrast-enhanced, dynamic CT acquisition ($CT_{pre}$, Toshiba Aquilion One), and two dynamic 3D transesophageal US images ($US_{pre}$ and $US_{post}$, Philips Medical System IE33) were acquired. $US_{post}$ data were not available in one patient. The voxel resolution values of the five datasets for both acquisition methods are reported in Table 1.

#### 3.1.1. CT/US Temporal Registration

In the first registration step, both CT and US images were temporally registered to improve registration results and optimize the processing time. CT images were acquired using a contrast medium and a retrospective ECG gating, which allows us to obtain different CT volumes at 10 specific phases in a complete cardiac cycle. US images were provided in a 4D (3D+T) volume continuously acquired from one or more cardiac cycles. Since US data acquisition was not synchronized with ECG, a frame selection step was necessary. The temporal registration step was performed using Python scripts [32] due to some limitations of 3D Slicer regarding the input data format and lack of specific time-registration modules. In this way, the development of a semi-automatic procedure (Figure 1) was possible.

**Figure 2:**
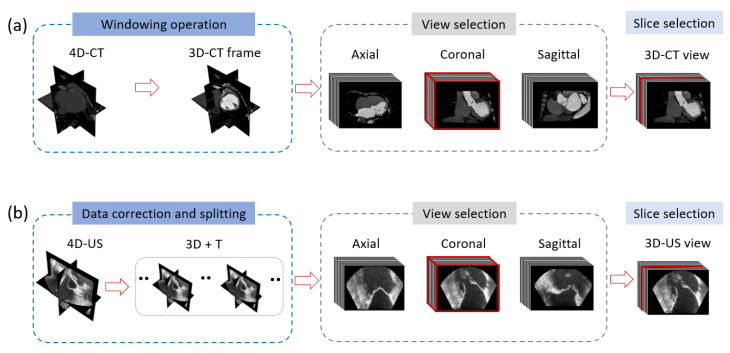
Workflow of the frame reference extraction operation for the CT (**a**) and for US (**b**): first, one volume view is selected; then, one slice is extracted from the CT/US volume selected view.

**Figure 3:**
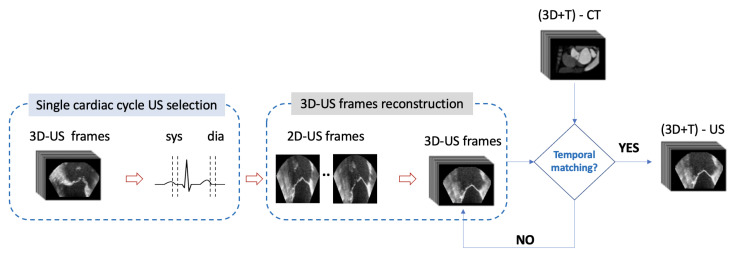
Workflow of the US/CT frames temporal registration.

First, the CT reference volume was chosen and displayed in tri-planar mode, selecting in the coronal view of the slice with the best view of MV (Figure 2a). The same procedure was followed for US volumes (Figure 2b). These steps were carried out in order to obtain the best match between CT and US images. For US frame selection, an entire cardiac cycle was extracted from US sequence based on the visual assessment of the MV movement, selecting the interval between two “fully open” states. In this way, the frames corresponding to the whole MV dynamic in a cardiac cycle were selected. Finally, the US frames that temporally matched with the CT frames were defined by a semi-automatic approach, matching the relative positions of CT and US frames in the cardiac cycle. Once the final US image frames were chosen, each frame of the corresponding volumes was reconstructed into a full 3D volume to be loaded in 3D Slicer (Figure 3).

#### 3.1.2. CT/US Spatial Registration

The CT-US spatial registration procedure was preceded by two important steps: interpolation and volume centering. The first operation was mandatory to make both data have the same spatial resolution. The software tool used for this purpose was the 3D Slicer “Resample Scalar Volume” module. Regarding the resampling, it was not trivial to choose the fixed and moving volumes in the procedure due to the presence of anisotropic voxels. In the end, the moving volume was chosen as the one that had a voxel size in one spatial direction much greater than the other. Therefore, CT data were resampled given the z-direction pixel size was greater than the US data, even if the latter had the worst resolution along the x-y directions. Following this reasoning, the spacing parameters of the “Resample Scalar Volume” module were set to 0.342 × 0.342 × 0.37 mm, selecting a linear interpolation. In particular, linear interpolation was chosen over the other types of interpolation methods mostly because of the better processing speed of the procedure (e.g., 4 s for linear interpolation vs 8 s for B-spline). Qualitatively, all the main interpolation methods were comparable. The second task was the CT volume centering which consisted of aligning the coordinate system origin of the CT volume with that of the 3D Slicer. The centering task was performed using the 3D Slicer function “Center Volume” in the “Volumes” module. Lastly, CT-US registration was carried out. Three-dimensional Slicer has several registration modules implementing automatic, semi-automatic, and interactive approaches (Table 2).

Since cardiac images rely mainly on soft tissues, warping methods are often used to compensate for anatomic deformations (i.e., cardiac activity in heart tissues) or patient movements (i.e., patient breath). However, non-rigid algorithms tend to significantly warp anatomic structures during the registration procedure, distorting the real structures and causing inaccuracy in the procedure assessment at the end of the registration pipeline. Therefore, the rigid transformation was considered the most appropriate technique, even if it came with inevitable image alignment mismatches. As an initial attempt, the automatic approach was first tested using the “General Registration (BRAINS)” module. All “Initialize Transform Modes” were tested. Only the “no initial transform” and the “moments align” modes converged. As expected, the results were poor and not accurate, presenting visible misalignment of the two volumes. Moreover, the time consumption of the procedure was proportional to the percentage of fixed volume voxels sampled for the registration. As an example, with 50% of voxel the tool converges after 10 min with a misplaced orientation regarding the fixed volume. So the automatic methods provided by 3D Slicer and applied to these data were considered unsuitable for achieving the desired outcome. Therefore, the semi-automatic approach was conducted after a comparison of the two 3D Slicer modules listed in Table 3. Given its features and advantages, the “Fiducial Registration Wizard” module was finally chosen. Fiducial points were placed all over the tri-planar views of both volumes, matching points were also placed between non-corresponding views. Points were evaluated by focusing mainly on the anatomical regions of the target of interest, such as the flaps and annulus of the MV, but also on characteristic points in the surrounding areas, such as the left atrium/ventricle or aortic root landmarks.

#### 3.1.3. Landmark Registration Optimization

A crucial aspect of the procedure concerned the number of landmarks necessary to obtain optimal registration. To meet this need, a validation of the registration method was carried out to assess both the accuracy and reproducibility of the obtained results. Therefore, intra-observer and inter-observer reproducibility validation was performed. The intra-observer and inter-observer reproducibility was assessed by carrying out 10 registration trials repeated by the same user or by two different users on two representative image datasets. In each test, a minimum number of pairs of fiducial points equal to 3 and a maximum equal to 12 was established. A reference transformation based on manual registration was defined as GS. Three types of errors were defined as metric functions to determine the optimal number of fiducial points based on the RMSE. RMSE1 index was evaluated as RMSE between the landmark transformation matrix parameters and the GS ones (Equation (1)).

(1) $$\mathrm{RMSE}1=\sqrt{\frac{1}{n}\sum_{i=1}^{n}{\left(M_{i}-MGS_{i}\right)}^{2}}$$

where *n* represents the total number of data points; $M_{i}$ and $MGS_{i}$ are the values of the evaluated registration and GS transformation matrix elements respectively.

The FRE was defined as reported in Equation (2):

(2) $$\mathrm{FRE}=\sqrt{\frac{1}{n}\sum_{i=1}^{n}(R{\left(x_{1}-y_{i}\right)}^{2}}$$

where *n* represents the total number of data points, $x_{i}$ and $y_{i}$, are the coordinates of landmark points after the registration R. FRE was automatically computed by 3D Slicer between the placed points on registered CT and the US volume.

Finally, RMSE2 (Equation (3)) was defined as the RMSE between two sets of landmarks: the “transformed fiducial” and the “GS fiducial”. The “transformed fiducial” represents the landmarks on the moving volume (CT) that underwent various transformations to match the corresponding US landmarks. These transformations might include translation, rotation, or scaling. On the other hand, the “GS fiducial” refers to the landmarks on the same moving volume, but on which the “GS transformation” was applied.

(3) $$\mathrm{RMSE}2=\sqrt{\frac{1}{n}\sum_{i=1}^{n}{\left(tf_{i}-gsf_{i}\right)}^{2}}$$

where *n* represents the total number of data points; $tf_{i}$ and $gsf_{i}$ are the coordinates of transformed and GS fiducial points, respectively.

The optimal landmarks number N was chosen as the number that guarantees the most accurate registration by minimizing the previously defined indices. As accurate anatomic positioning of later landmarks becomes more difficult when increasing N, and the required registration time increases with N, the best N value represents a compromise between speed and registration accuracy. The best N value was chosen by verifying intra-observer and inter-observer reproducibility values across the whole study population, as will be detailed in the following. Finally, to understand whether the obtained results were valid, in addition to a visual evaluation of the images, a numerical comparison was also made with the typical dimensions of the anatomical target of interest (i.e., the MV annulus).

### 3.2. Case Study 2: Landmark Registration in Orthopaedics

**Figure 4:**
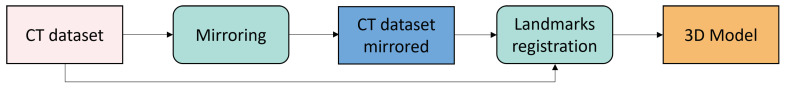
Workflow of the orthopaedic registration.

The datasets used in this case study were two different CT acquisitions (Philips Medical System) of the skull, each with specific dimensions and pixel spacing. The first dataset measured 476 × 960 × 173 with a voxel size of 0.32 × 0.32 × 0.62 mm, while the second dataset had dimensions of 476 × 960 × 219 and a voxel size of 0.81 × 0.81 × 1.00 mm. Both data depict the skulls of maxillofacial reconstruction surgery patients. In this case study, our goal was to develop a patient-specific procedure for generating a 3D reconstruction of the affected portion of the jaw that required replacement. The procedure involved several steps to ensure an accurate and personalized reconstruction, as illustrated in Figure 4.

The initial step focused on segmenting the patient’s CT dataset using the 3D Slicer software. This segmentation process aimed to obtain a virtual 3D reconstruction of the jaw and the surrounding areas of interest. A threshold segmentation technique was employed to identify the jaw and other bone structures, while the tumour mass required threshold segmentation with manual correction. These segmentation techniques allowed for the precise delineation of the relevant anatomical structures. The second step of the procedure involved mirroring the healthy side of the jaw onto the damaged side to reconstruct the abnormal area. This was achieved by digitally mirroring the CT dataset using the sagittal plane as the mirroring axis, passing through the centre of the jaw. By mirroring half of the CT volume, which included the healthy side, onto the affected side, a template for the reconstruction was created based on the shape of the healthy jaw side. This step was performed with a specific Python code. However, a comparison between the original and mirrored datasets revealed misalignment.

To address this misalignment, the third step of the procedure involved local registration of the mirrored dataset with the original dataset, specifically focusing on the region with the part of the mandible requiring replacement. This local registration process was performed within the 3D Slicer software, utilizing the landmark registration module. Landmarks were placed in tactical anatomical areas adjacent to the mandible to be reconstructed but unaffected by the tumour mass. A rigid registration approach was chosen for its simplicity and effectiveness in aligning the mirrored and original datasets within the defined region. This final step allowed for better matching with the original anatomical geometry.

## 4. Results

### 4.1. Case Study 1

**Figure 5:**
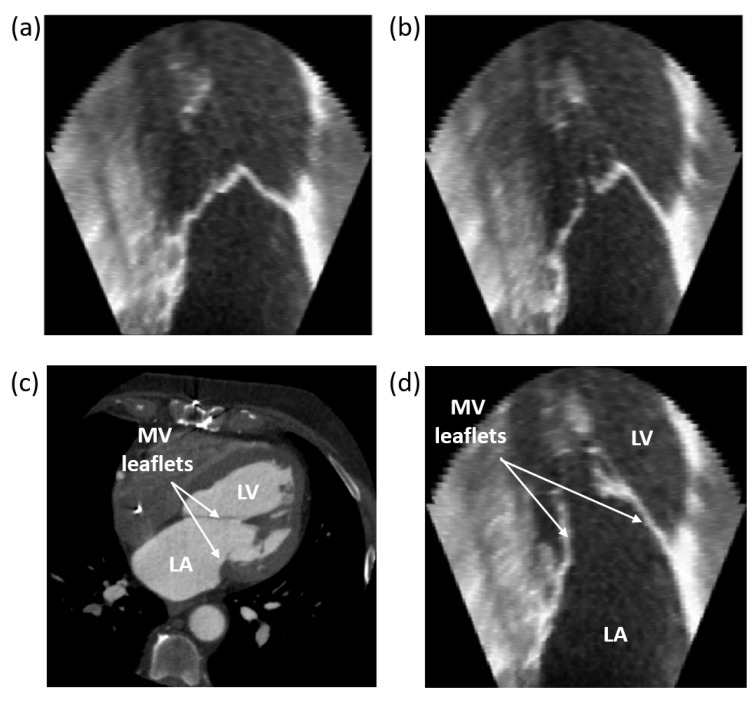
Results of the US frames selection related to one cardiac cycle. Frame selection related to the beginning (**a**) and to the end (**b**) of the cardiac cycle. The CT (**c**) and US (**d**) frame comparison operations approximately match in time, as confirmed by the MV leaflets opening in both volumes.

In terms of the CT/US temporal registration results, an example of the temporal registration between CT and pre-operative US data is reported in Figure 5. After selecting the 10 temporal frames constituting the CT volume, the US reference temporal frame and the reference image were chosen (Figure 5a,b). Then, the US reference image most similar to the CT one was selected, choosing among the slices belonging to the US volume on the coronal view (Figure 5c,d).

**Figure 6:**
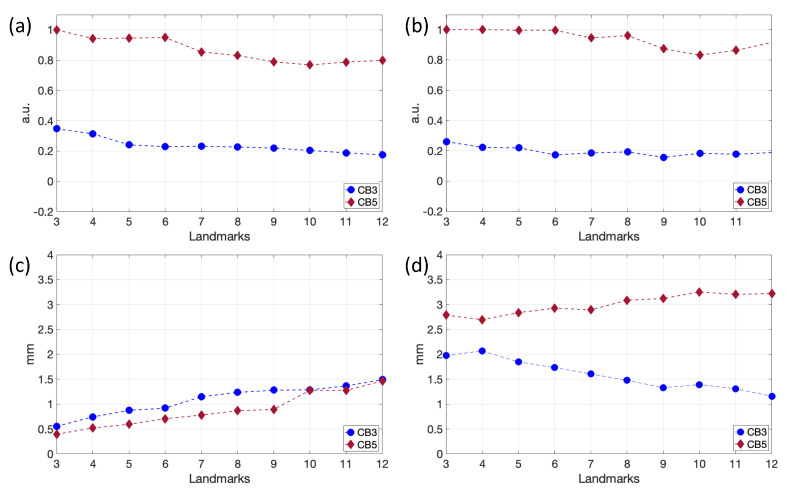
RMSE 1 of rotation (**a**) and translation (**b**) parameters plots averaged over the number of trials, as the number of pairs of landmarks varies. FRE (**c**) and RMSE2 (**d**) plots for CB3 and CB5 of User1, averaged over the trials, as the number of pairs of landmarks varies.

In terms of the landmark registration optimization results, after the temporal alignment of CT and US data, 3D Slicer was used to proceed with spatial registration. First, one volume for each imaging modality corresponding to 90% of the cardiac cycle (end-diastole) was loaded. Based on the resolution of the images (Table 1), the CT and US volumes were first interpolated and then centred. Three-dimensional Slicer was used to perform a rigid landmark registration procedure, choosing fiducial points by looking for the most visible anatomical landmarks between the various views and slices of the two volumes. As described in Section 3.1.3, to find the optimal landmarks number necessary to obtain acceptable registration results, the registration method was validated. First of all, intra-observer reproducibility verification was performed based on RMSE and FRE errors computed on two different datasets, namely CB3 and CB5, over 10 trials. In Figure 6, for both patients, the trends related to RMSE1 for both rotations and translations parameters and the trends in the FRE and RMSE2, averaged over the trials, are reported as the number of pairs of landmarks increases.

The RMSE1 values related to the rotation (Figure 6a) and translation (Figure 6b) matrices’ parameters show a slight decrease as the number of pairs of landmarks used increases, with the same trend for both patients. The minimum RSME1 value was reached at 10 and 12 landmarks for patient CB5 an CB3, respectively. As transformation matrices were obtained from 3D Slicer, which performs implicit concatenation of matrices, numerical values of the RMSE1 index are not significant and were expressed in arbitrary units.

The FRE values (Figure 6c) show a progressive increase as the number of landmarks increases for both patients. This finding can be explained by the fact that the first landmarks are placed in the most evident anatomical regions, so, the more landmarks are inserted, the more difficult is to position them correctly. Finally, the RMSE2 values (Figure 6d) related to the transformed and GS fiducials points show a different trend in the two patients. A better registration quality was assessed in patient CB3. Registration quality increases with landmark number in patient CB3 and decreases in patient CB5. A possible explanation is that the better image quality in patient CB3 allowed for more effective landmark placement with respect to patient CB5.

**Figure 7:**
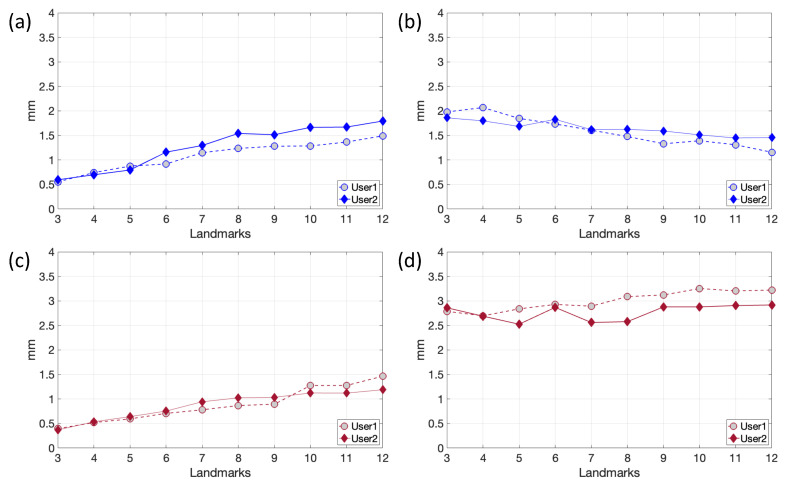
Inter-operators comparison of the FRE (**a**,**c**) and RMSE2 (**b**,**d**) plots for patients CB3 (**a**,**b**) and CB5 (**c**,**d**), averaged over the trials, as the number of pairs of landmarks varies.

To further verify the previously assessed FRE relation with a good registration result, an inter-observer reproducibility validation was then carried out (Figure 7).

The inter-observer results show good reproducibility with similar curves for both users. Obtaining lower FRE values does not necessarily mean performing good registration and vice versa. In fact, the value of these errors depends both on the number and on the careful choice of the anatomical landmarks used. Surely, less significant anatomical points describing the target lead to higher FREs, increasing the chances of picking the wrong position for the fiducials. On the other hand, more fiducials mean having high FREs since fewer landmarks are visible and more time is spent positioning them (thus increasing the chances of making more mistakes).

**Figure 8:**
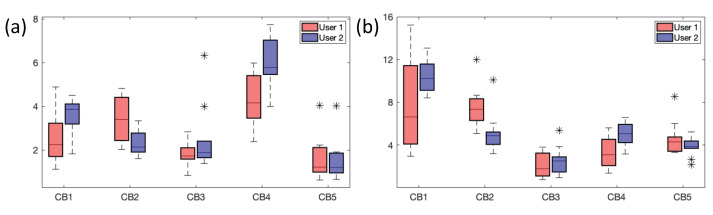
Box plots o f the FRE (**a**) and RMSE2 (**b**) values computed on 10 landmarks over 10 trials and related to the two users.

Based on the above observations, a total of 10 pairs of landmarks was chosen and tested by verifying intra-observer and inter-observer reproducibility on the whole patient population (Figure 8). In particular, the box plots show the FRE (Figure 8a) and RMSE2 (Figure 8b) values obtained on all patients by the two users. A good agreement between users was found for FRE values. The only exception is represented by patient CB4 in the User2 analysis (Figure 8b), where the median registration error is slightly higher than values in other patients. Regarding RMSE2 values, a slight discordance was found in patients CB1 and CB2. In the User1 analysis, patient CB1 appears to have a wide dispersion and variability of the measures, while patient CB2 has the highest median of the values.

**Figure 9:**
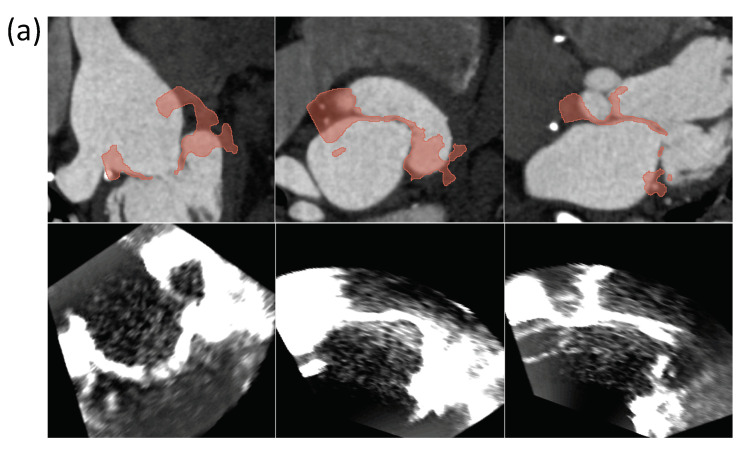

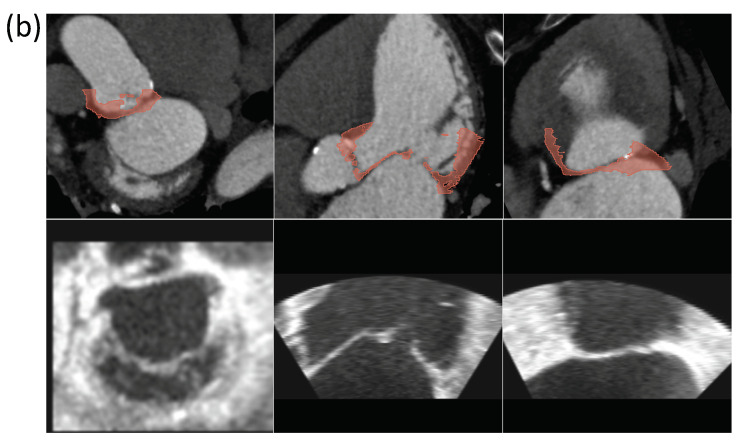
The CT/US registration results for patients CB1 (**a**) and CB2 (**b**) in axial, coronal, and sagittal view. The upper row shows the overlapping of CT and MV segmentation from the US for each plane. The bottom one shows the respective US planes.

Overall, the effect of correctly positioning the fiducial points is noticeable, which strictly depends on the type and quality of the examined patient. For instance, in patient 1, low FREs but high errors in GS fiducials for both users can be seen. There are different possible reasons for this behavior. The US image dataset is poor in temporal frames, thus increasing the probability of making errors in the temporal matching with the CT images. Consequently, the user has more difficulty finding the correct correspondence of the anatomical landmarks. Also, the US image dataset orientation is very different from the CT one as the US probe is mobile and orientable in any direction; this leads to a mismatch in the views of the two modalities and consequent difficulty for the user in finding the correct correspondence of the anatomical landmarks. Finally, the fiducial points are not equally placed on all the views of volumes. Therefore, the transformation matrix will maximize the registration in some direction with respect to the others. Overall, the results of the registration errors operated by the two users on the five patients and shown in the boxplots in Figure 8 point out that all the errors are below the maximum RMSE and FRE values of 15 mm and 8 mm respectively. These values are comparable with the target size as reported in the literature [33,34,35,36,37]. Overall, the obtained results show that 10 pairs of landmarks can be considered enough to carry out a robust registration. *For the fusion of CT/US,* once the number of landmarks to be used was defined and suitably placed, the registration was carried out by applying the transformation matrix (i.e., the 3D Slicer landmark registration module output) to the US volume. In all five datasets, a satisfying MV leaflet overlapping for all three views was obtained. To also assess the accuracy of the registration process from a qualitative point of view, the overlap between the two patients’ CT and US is reported in Figure 9.

### 4.2. Case Study 2

**Figure 10:**
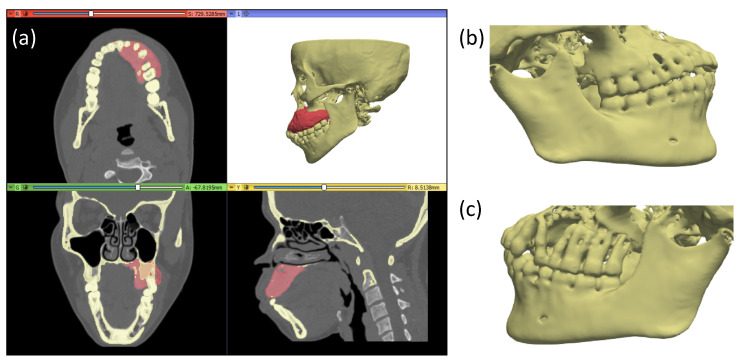
Three-dimensional models of the tumour mass and the jaw (**a**) and the detail of the healthy (**b**) and abnormal (**c**) side of the jaw.

The different segmentation techniques employed for the modelling of the jaw and the tumour allowed the generation of accurate geometries of the target anatomical structures (Figure 10).

**Figure 11:**
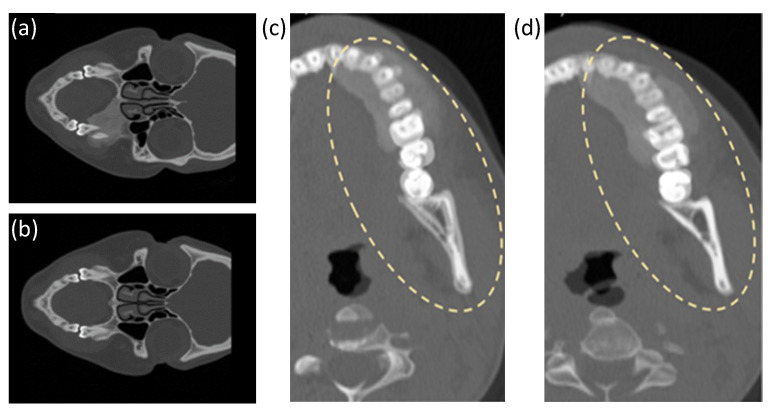
Mirroring pipeline from the original slice dataset (**a**) generates the mirrored slice dataset (**b**). The following registration procedure solves the matching gap visible in the overlapping of the pre-registered mirrored and original volume (**c**), as reported in the overlapping of the post-registered and original volume (**d**).

The mirroring operation (Figure 11a,b) leads to misalignment, as visible in Figure 11c, due to two factors: the imperfect symmetry of anatomical structures and the choice of the mirroring plane based on the CT acquisition planes, which might not always align perfectly with the anatomical structure of interest. The landmark registration module compensated for this issue, locally adapting the mirrored jaw to the original target (Figure 11d).

**Figure 12:**
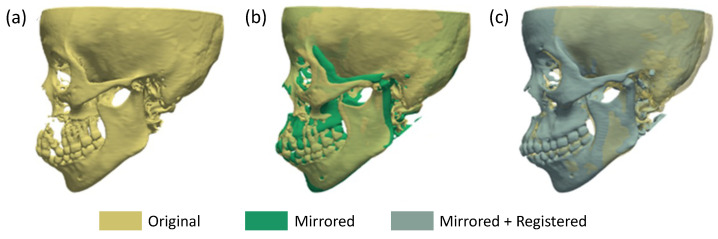
Three-dimensional model of the original dataset (**a**), overlapping of the original and mirrored dataset (**b**), and overlapping of the original and registered mirrored dataset (**c**).

Following the successful mirroring and registration steps, the new post-registered volume of data were obtained, representing the missing side of the jaw. This new dataset underwent segmentation using a threshold segmentation technique, with intensity values corresponding to those used for the segmentation of the original dataset. The resulting 3D reconstruction provided an accurate representation of how the missing side of the jaw (Figure 12a) should ideally be reconstructed starting from the original volume.

The combination of mirroring and registration led to a reconstructed 3D model (Figure 12c) that closely approximated the original jaw structure with respect to the only mirroring (Figure 12b), enhancing the potential for surgical guidance and successful replacement.

## 5. Discussion and Conclusions

In this paper, the implementation of a multimodal image registration procedure by using the free 3D Slicer software was discussed. Three-dimensional Slicer was chosen as it is a software tool which is genuinely useful for surgeons or physicians based on the following key features: open-source, access to images in DICOM format, a GUI, and image registration/fusion toolboxes. Other software platforms consistent with the above features are available, such as MITO (Medical Imaging Toolkit) [38], FW4SPL (FrameWork for Software Production Line) [39], and MediPy [40]. After testing the abovementioned software, 3D Slicer was chosen as the most suitable software to implement in the case studies.

In clinical practice, there has been increasing recognition of the potential of registration as a powerful tool to fuse information coming from different medical imaging techniques. In cardiology, clinical applications range from intra-operative image fusion, which represents the more challenging scenario, to pre-operative planning and the post-operative assessment of the intervention. The post-operative assessment of MV function from pre-operative CT and pre- and post-operative US is an example of a scenario where clinicians can fully take advantage of software registration tools to improve the procedure accuracy. In particular, the developed procedure could allow the quantification of the improvement in MV function obtained by the interventional procedure. The study results showed the importance of a pre-elaboration of the data to increase the accuracy of the registration, ensuring a good temporal matching. Indeed, temporal registration was implemented for the CT and US images, which were temporally synchronized. An interactive operation flow allowed to find, in a semi-automatic way, the temporal correspondence between US volumetric frames and CT ones, and the registration process was carried out by choosing two frames (one for each imaging modality) belonging to the same cardiac phase. After the temporal registration, the spatial registration was carried out using the landmark registration module. To assess the registration accuracy, two key metrics were selected: the RMSE and the FRE. Remarkably, in both cases, the maximum RMSE and FRE values were found to be as low as 15 mm and 8 mm, respectively. These values are notably small when considering the dimensions of the MV, indicating the robustness and precision of the registration process. The validation of the landmark registration, by determining the number of fiducial points to use to have good accuracy, showed a critical dependency of the procedure on different factors. The increase in the number of points leads to an increasing registration error. This is linked to the limited number of significant anatomical landmarks in the target and the quality of the fixed and moving images. Moreover, different imaging modalities have different acquisition orientations for the same target, leading to matching points being positioned in mixed planes other than the standard ones. All these factors were derived from previously discussed statistics that helped us in fixing a reasonable number of fiducial points.

In the current literature, according to the authors’ knowledge, the only similar workflow and results were found in Rahimi et al. [41], where, using MATLAB software, a registration between three different imaging modalities (CT, MR, and US) was implemented for a thoracic valve implantation and replacement procedure. In this case, an initial time-matching step between the different modalities is defined, followed by spatial registration based on normalized mutual information maximization. Differently from our work, registration was performed on 2D slices, and result validation was based on Dice score and Hausdorff distance (HD) metrics evaluated on manual segmentations performed by experts. Hence, the error values presented in the current study cannot be directly juxtaposed with those of the Rahimi study, which reports an HD value of 1.49 ± 0.20 mm. Several studies about bimodal CT/US registration are present in the literature. Differently to the present study that involves 3D-3D registration, these studies employ 2D-2D [42] or 2D-3D [43,44] registration. In their investigation, Huang et al. [43] disclosed a Target Registration Error (TRE) of 1.7 ± 0.4 mm while aligning 2D US images with cardiac 3D CT images using a phantom. Furthermore, Lang et al. [42] devised a registration framework to match 2D US with 2D CT scans, resulting in a TRE of 1.5 ± 0.45 mm, specifically for the aortic root. In contrast, Khalil et al. [44] introduced registration frameworks for US to CT and indicated a TRE of 1.32 ± 0.04 mm, specifically for the aortic valve. It is noteworthy that, besides not registering directly 3D volumes, these studies integrated optical tracking systems into their registration frameworks, except for [41,44]. Despite a higher error in the registration process concerning other methods in the literature, the current method’s results are achieved without the integration of tracked systems and working directly on 3D volumes for both US and CT data. Hence, the proposed work demonstrates the use of a free software tool, 3D Slicer, as an affordable and efficient registration tool that can be used with acceptable performances in a clinical setting. The procedure, validated on five patients in MV registration, was tested on a second case study in a different clinical field, focused on the pre-operative planning of jaw reconstruction. After a mirroring operation of the starting data regarding the centerline volume, the same landmark registration module allowed for a local registration of the reconstructed jaw on the original anatomy of the patient. From previous work in the literature, the mean linear differences regarding the accuracy in predicting maxillary positioning are typically within the range of 2 mm [45,46,47,48,49,50]. In the majority of these investigations, accuracy was evaluated by measuring the mean linear difference between specific reference points. In addition, accuracy can also be evaluated by assessing the linear difference between superimposed surfaces, as in Tucker et al. [50]. Moreover, it is worth saying that the jaw registration is here reported as proof-of-concept on the feasibility to use 3D Slicer tools into different clinical scenarios. For this reason, quantitative results are not provided for a comparison with the existing literature. However, it is important to note that the described procedure is specifically applicable to cases where the damage to the jaw is limited to one side only. In situations where the anomaly affects both sides of the jaw, the mirroring process alone is insufficient for the complete reconstruction of the missing parts. As a result, the reconstruction using the above-described procedure may yield an incomplete jaw model, which does not provide adequate guidance for the replacement procedure. One potential approach is to integrate the segmented model with a computer-aided design reconstruction of the missing parts based on reference anatomical atlases. Indeed, for cases involving extensive damage to both sides, further refinements and integration with computer-aided design reconstruction may be required to ensure a complete and precise jaw replacement. Integrating multiple data sources can help us to achieve a more comprehensive and accurate reconstruction, enabling surgeons to better plan and execute the replacement procedure. Overall, the combination of mirroring, registration, and segmentation techniques improved the accuracy and applicability of the reconstruction process, leading to more successful outcomes in cases where the damage is limited to one side of the jaw.

Although the proposed workflow has provided satisfactory registration results, one of its limitations consists of using external software for the execution of some tasks (i.e., the temporal registration procedure). Since the goal is to use this procedure for clinical use, it would be convenient for the clinician to only familiarise himself with a single platform to simplify the registration operations and speed up execution times. For this purpose, a specific 3D Slicer module could be developed to integrate these operations. Moreover, future work will consist of increasing the number of considered patients in order to further strengthen the results already obtained. Finally, another future development could be making the registration workflow adaptable to other surgical scenarios as well, trying to guarantee performances such as those obtained in the surgical context treated in this paper. In orthopaedic settings, making jaw reconstruction and registration consistent with the previous anatomical target is a challenging task. This paper offers a possible fast and straightforward solution to the problem by using 3D Slicer.

In conclusion, in this paper, multidomain registration case studies in both mono and multi-modality were explored in both the cardiovascular and orthopaedic fields, highlighting the benefits and challenges associated with their implementation in a clinical setting. The proposed workflow demonstrated effectiveness in the assessment of mitral valve function after repair. The developed procedure performed well also in a single case of planning of orthognathic procedure. By understanding the potential of multimodality registration, clinicians can make full use of the medical information it provides to advance patient care and improve outcomes. 

## Figures and Tables

**Table 1:** Image resolution for $CT_{pre}$, $US_{pre}$, and $US_{post}$ in all five patients.

Patients	*CT_pre_*		*US_pre_*		*US_post_*	
	Spacing (mm)	Dimensions	Spacing (mm)	Dimensions	Spacing (mm)	Dimensions
CB1	0.47 × 0.47 × 1.00	512 × 512 × 160 × 10	0.50 × 0.50 × 0.27	208 × 192 × 208 × 19	0.59 × 0.58 × 0.41	208 × 176 × 208 × 13
CB2	0.40 × 0.40 × 1.00	512 × 512 × 160 × 10	0.58 × 0.57 × 0.37	208 × 192 × 208 × 13	0.99 × 0.99 × 0.64	144 × 144 × 208 × 42
CB3	0.47 × 0.47 × 1.00	512 × 512 × 160 × 10	0.51 × 0.50 × 0.27	256 × 240 × 208 × 52	**	**
CB4	0.47 × 0.47 × 1.00	512 × 512 × 160 × 10	0.55 × 0.55 × 0.31	208 × 208 × 208 × 10	0.50 × 0.50 × 0.13	208 × 208 × 208 × 36
CB5	0.47 × 0.47 × 1.00	512 × 512 × 140 × 10	0.47 × 0.47 × 0.22	192 × 208 × 208 × 12	0.51 × 0.51 × 0.29	208 × 160 × 208 × 11

** Image data not available.

**Table 2:** List of 3D Slicer image registration tools evaluated.

Automatic General Registration	Semi-Automatic	Interactive
BRAINS	Landmark Registration	Transforms
ANTs	Fiducial Registration Wizard	
Elastix		

**Table 3:** Main features of the 3D Slicer semi-automatic registration modules evaluated.

3D Slicer Module	Features
Landmark Registration	Only suitable for similar volumes
	Automatic landmarks placement
	Rigid and warping transformation types supported
Fiducial Registration Wizard	Suitable similar and dissimilar volumes
	Manual landmarks placement
	Rigid and warping transformation types supported
	Transformation matrix and registration error (RMSE)

## Data Availability

The data presented in this study are available on reasonable request from the corresponding author.

## Abbreviations

The following abbreviations are used in this manuscript:

CT	Computer Tomography
MRI	Magnetic Resonance Imaging
US	Ultrasound
SSD	Sum of Squared Differences
MI	Mutual Information
TAVI	Transcatheter Aortic Valve Implantation
PET	Positron Emission Tomography
MV	Mitral Valve
NMI	Normalized-MI
CNN	Convolutional Neural Network
$CT_{pre}$	Pre-Operative CT
$US_{pre}$	Pre-Operative US
$US_{post}$	Post-Operative US
GS	Gold Standard
RMSE	Root Mean Square Error
FRE	Fiducial Registration Error
TRE	Target Registration Error
HD	Hausdorff distance
